# Promoting an Understanding of Forced Migration Among Host Country Children and Exploring Their Views on Refugee Children’s Needs

**DOI:** 10.1007/s10903-022-01395-9

**Published:** 2022-08-24

**Authors:** Anna Sarkadi, Elin Lampa, Georgina Warner

**Affiliations:** Child Health and Parenting (CHAP), Department of Public Health and Caring Sciences, Uppsala University, Box 564, BMC, Husargatan 3, Uppsala, Sweden

## Background

We know that refugee children are vulnerable to develop mental health problems, particularly post-traumatic stress [1]. Yet, social relationships including the number of friends and quality of friendship appear to play an important protective role [2]. Feeling accepted and supported by peers reportedly promotes wellbeing among refugee children [3, 4], and is associated with lower levels of psychological distress [4], emotional difficulties [5] and aggression [6]. Conversely, perceived discrimination by peers is related to emotional problems and aggressive behaviour [6]. Given the significant role peers play for refugee children, efforts should be made to engage host country children with the topic of refugee children’s needs. This brief report describes a film-based activity carried out at a children’s science festival in Uppsala, Sweden. Uppsala is a university town with a general education level higher than the national average, but also with less affluent areas from which schools visited the science festival. A short film conveyed a typical experience of a refugee child to provide the children with contextual knowledge and a qualitative survey was used to collect the children’s views on what they think refugee children might need when they arrive in a new country. The qualitative design was selected to give the children space to provide detail about their reasoning in their own words and to enable the nuanced perspectives of children across various ages to be captured. As the survey was explorative, there were no specific hypotheses.

## Methods

### Film

‘Ali and the Long Journey to Australia’ is a clay-animated short video of the experiences of a refugee child. “Ali” is a 10-year-old boy who tells his story from a bomb attack destroying his house, to police coercion against his father, through the dangerous boat journey across the sea, his stay in a refugee camp, the insecure time awaiting asylum, and finally being able to go to school. The story ends better than for many refugees: the family reunites in the new country and can start their life there together. Originally developed as a book, the video was co-produced with 13 primary school pupils in South Melbourne, Australia. With permission from the developers, we translated the video content into Swedish and created a Swedish-speaking child voice/over for the film.

### Participants

A total of 51 children completed the activity. They were approached as they passed the research group’s stand at the science festival. The only inclusion criterion was being under 18 years old. The majority of approached children were willing to participate and all of these were included in the study. Of the children who took part, the age range was 5 to 14 years (mean = 9.6, SD = 2.2). About half of the respondents were boys (n = 26) and half were girls (n = 23); two respondents did not wish to state their gender. When asked if they knew someone like Ali, 14 (27%) stated that they did, 27 (52%) said they did not, 10 (19%) were not sure, and one child did not respond.

### Data Collection

The film was available on tablets for the children to watch. A Swedish translation of the book was also available at the stand. The science festival attendees were made aware that they would be asked to complete a brief anonymous survey after watching the film, that we were a research group who are interested in what children have to say, and that we would like to write about the findings. Swedish legislation mandates that ethical clearance is not required for anonymous studies, including studies with children. All children and parents were asked for oral consent on site, but without registering their names or any other personal identifying information. There was always a qualified adult (psychologist, nurse or physician) there to assist them if questions or difficult emotions arose. Parents of children under 9 years of age were informed that the film had some difficult content and that parental presence was advised, as well as watching the whole film, given the positive outcome.

The children were asked to complete the anonymous survey on the tablet directly after watching the film. Besides their age, gender, and whether they knew someone like Ali, we asked the children: (i) What do you think newly arrived children need in Sweden? and (ii) How do you think we could help them with their needs? The readability of the questions was categorized as ‘very easy’ according to the Läsbarhetsindex Swedish Readability Formula [7]. Most children typed directly into the survey form, but some (mainly younger) children requested for the research group member to help; this resulted in an interview format for some children, which involved the researcher reading the question then typing the child’s response into the survey form verbatim.

### Analysis

The responses were extracted from the online survey platform into Excel and translated into English before being analyzed. Data from the open-ended questions were combined and analyzed using content analysis [8]. Initially, the manifest content was identified for each answer and categorized. The category coding was conducted by two independent raters. The inter-rater reliability was (i) κ = 0.95 (p < .000), 95% CI (0.88, 1.02) and (ii) κ = 0.89 (p < .000), 95% CI (0.8, 0.98) for the respective questions. The following step included identifying the latent content, i.e. the meaning behind the statement. In some cases, the answers were too short to allow for latent content analysis – for these, only the manifest content was identified. Additionally, a quantitative comparison on the code level was made between the children who stated they knew someone like Ali, and those who stated they did not know someone like Ali.

## Results

Four latent categories emerged in the analysis: ‘Practical support’, ‘Emotional support’, ‘Social inclusion’, and ‘Policies’ (see Table 1). A developmental trajectory in the nature of responses was observed, with the older children providing more complex responses. No meaningful differences were observed between genders nor the responses from children who reported knowing someone like Ali and those who reported not knowing someone like Ali.

**Table 1:** Overview of findings

Category	Description	Example quote(s)
Practical support	Responses in this category focused on the urgent and everyday needs of refugees, such as getting access to housing, food, and clothing. ‘Home or House’ was the most common need mentioned, but many children identified several of the basic needs. Children mentioned ‘school’, recognizing education as a fundamental need. A number of children identified that learning the Swedish language was among the practical support that would be required by newly arrived children. The youngest children’s answers were dominated by these practicalities, but they were also the most prevalent answers given overall.	*“One could give them a home, food, and love” (girl, 12)* *“School, own house, food, friends” (boy, 12)* *“They need help to learn our language” (girl, 6)* *“Food, toys, water, breakfast” (boy, 6)*
Emotional support	The manifest content included in this category covered expressions of kindness and love. Their answers demonstrated empathy and concern for the refugee children. The children talked about the need to be met with compassion and understanding in the host country, with many of them expressing that peers should “be kind”, “be good to them” and offer them “warmth” and “love”. In addition, some children demonstrated an awareness of the psychological needs of the newly arrived children; that they may be traumatized by the forced migration experience and need to talk about it, but that they should not be rushed to do so.	*“Don’t ask too many questions. Let them tell about what happened. Let them digest what has happened” (girl, 11)* *“Be kind” (boy, 7)* *“Protection and warmth” (girl, 11)* *“Have a friend who understands” (boy, 10)*
Social inclusion	This category included positive examples of how to socially include the newly arrived children, including “play with them”. A few children also highlighted the potential threat to social inclusion of being bullied, as the newly arrived children may not have the same possessions or language as their peers. Bullying was mentioned by the children in a negative tone, expressed as something that should be deterred. Others spoke of social inclusion in very general terms, comparing refugee children with peers and expressing that all children should be given the same opportunities.	*“Not be bullied or teased because one doesn’t have what others have” (boy, 12)* *“See and treat them like an ‘ordinary’ child… give them the same possibilities as for the other children” (girl, 14)* “Play with them” (boy, 9)
Policies	Within this category, some responses were specific in how Swedish society could support newly arrived children. For instance, several children recognised that resources are needed for helping refugees and suggested using tax money to provide help, or donating to non-governmental organisations that provide support and aid to refugees. Other children made more general comments that related to immigration policy, such as “let them in” or speaking of the need to provide “protection” to the newly arrived children, which inferred a need to preserve their civil liberties and rights.	*“A little part of the tax could go to them” (boy, 10)* *“They need help from the Swedish society - let them in.” (boy, 8)* *“Donate money to organisations that help them” (girl, 11)*

## Discussion

The children who completed the science festival activity rightly recognized that in the initial stage of arrival, practical resources, such as a home, food, clothes, and toys are important to alleviate the adaptation to the new context, which corresponds to the basic needs of all humans. This indicates the children were aware of the refugee narratives in the media at the time. The answers were also likely influenced by the film, where Ali and his family had lost their home and then all their belongings when their boat capsized at sea.

It is interesting that (even younger) children seemed to intuitively recognise the rather immediate need for friendship and kindness, i.e. social support and inclusion. Given the protective nature of peer support [3–6], it is encouraging that words such as “help” and “love” were used. Some respondents also mentioned the need for protection in general, and specifically from bullying or discrimination. Again, this is reassuring given the detrimental effect of perceived discrimination on wellbeing [6].

It is intriguing that rather young children shared comments recognizing the connection between policies and individual refugee children’s needs. We know that policies and public attitudes do matter for refugee children. For instance, in countries with supportive multicultural policies school belonging is higher whereas restrictive policies reportedly limit the school belonging of students [9].

## Limitations

We acknowledge the content of the video may have influenced the subsequent answers about the needs of refugee peers. Utilising a survey-based response method resulted in relatively brief responses, but was fitting to the community engagement style of the interaction with the children and offered greater anonymity to encourage honesty. A relatively small group of children was involved in the activity and participation was voluntary. Yet, the code saturation method [10] indicated saturation was achieved. To ensure the credibility and confirmability of the study, the first author (AS) conducted the analyses and the second and third author (EL, GW) independently reviewed the manifest and latent categories. The dependability of the findings was enhanced by thorough documentation of the data collection and analysis process. Further information, such as migration background, ethnicity/cultural background, social class, and family constellation could have been helpful in interpreting the results but the brevity of the survey form was prioritized.

## Conclusion

The findings from this study indicate the capability of children in Sweden to consider the needs of refugee children and suggest that resources such as ‘Ali and the Long Journey to Australia’ could be potentially helpful in fostering an inclusive school environment. Other resources to teach children about forced migration are freely available, such as those produced by United Nations High Commissioner for Refugees (unhcr.org/teaching-about-refugees), and should be considered by school personnel. Yet, awareness raising among peers only forms one part of the picture. To create an inclusive environment for children who have experienced forced migration, an equity-focused and trauma-informed approach across school culture, practice and policy is encouraged. This should also include highlighting the strengths and aspirations of children from refugee backgrounds, not only their needs. We recommend further exploring children’s views and ideas through qualitative enquiry.
